# An IoT System Proposed for Higher Education: Approaches and Challenges in Economics, Computational Linguistics, and Engineering

**DOI:** 10.3390/s23146272

**Published:** 2023-07-10

**Authors:** Liana Luminița Boca, Elisabeta Mihaela Ciortea, Carmen Boghean, Andreea Begov-Ungur, Florin Boghean, Vasile Teodor Dădârlat

**Affiliations:** 1 Department of Informatics, Mathematics and Electronics, Faculty of Computer Science and Engineering, “1 Decembrie 1918” University of Alba Iulia, 510009 Alba Iulia, Romania; mciortea@uab.ro; 2 Computers Department, Faculty of Automation and Computers, Technical University of Cluj-Napoca, 400027 Cluj-Napoca, Romania; vasile.dadarlat@cs.utcluj.ro; 3 Department of Economics, Economic Informatics and Business Administration, Faculty of Economics, Administration, and Business, “Ștefan cel Mare” University of Suceava, 720225 Suceava, Romania; carmen.boghean@usm.ro; 4 Department of Cadastre, Civil Engineering and Environmental Engineering, Faculty of Computer Science and Engineering, “1 Decembrie 1918” University of Alba Iulia, 510009 Alba Iulia, Romania; andreea.begov@uab.ro; 5 Department of Accounting, Audit and Finance, Faculty of Economics, Administration, and Business, “Ștefan cel Mare” University of Suceava, 720225 Suceava, Romania; florinboghean@usm.ro

**Keywords:** IoT, system, Petri nets, model, higher education, teaching

## Abstract

The technological revolution and the evolution of technology have significantly facilitated the applicability of the IoT in various domains, such as healthcare, transportation, agriculture, retail, education, and, especially, higher education, which encompasses countless areas. Petri nets can be a useful tool to model the behavior of an IoT system. The main objective of this paper was to propose, model, and analyze a complex IoT system for higher education. The system involves the integration of IoT devices for monitoring data. An educational cloud was used as a support tool through which tracking, and control actions were implemented both internally, between the cloud and entities, and externally, between the cloud and the IoT. The system was modeled using Petri nets, which are systems with discrete events, and for simulation, we used the Visual Object Net++ package. Using this application, information was obtained in real time, and it was possible to intervene with changes even in the design phase. The diagrams were easy to read and interpret, which is an advantage for the decision-making system. The general structure of the application was based on *n* entities, where each entity represented a higher education field. In this paper, we discuss at least three fields: economics, computational linguistics, and engineering.

## 1. Introduction

In the current context, revolution and technological evolution have completely changed mindsets, processes, and behaviors, and in some domains, the impact is more than significant. For example, in certain industries, almost everything has been mechanized, and processes are followed remotely via the Internet. This highlights the need to interconnect the devices used and to store data efficiently, often using the advantages offered by clouds.

Higher education, more than any other field, focuses on the teaching/learning side and on the study of the latest developments both in the technological and ideological areas. Nowadays, the IoT has become a permanent implicit concern in the scientific field, which creates an open field for interdisciplinary research.

In our research approach, we identified several important premises, as follows:

- Can IoT devices help to streamline the educational process?
- What would be the optimal way for the data used in the teaching–learning process to be accessible to all stakeholders, students, and teachers in real time?
- Is a system consisting of IoT devices and an educational cloud in which data from different domains are stored viable?

One of the major aims of this work was to create a system designed specifically for the educational environment that is based on the use of IoT devices for sending and obtaining data stored on a cloud dedicated to this purpose—a cloud accessible only to individuals who are part of the specific entities of the institution.

This paper is a heuristic presentation of a study on the educational system. In this paper, we wanted to highlight why this study is necessary and how modern technology can be used in a relatively easy way in the educational system [1]. Today, we approach the technologies used in education with access to several entities and a cloud for managing and viewing information on IoT devices. Through the approach of advanced methods of analysis and learning of technological equipment, hardware systems can adapt to the fluctuations of the work process over time. According to the literature, this method has been known of and developed for several years; however, the method is mainly used in researching and tackling IoT modules. Recently, such approaches have begun to be accessed in education, but with empirical studies. The IoT uses evolving technologies with advanced cloud-based software to produce large amounts of real-time data [1].

The Petri nets used in the paper for real-time system modeling and simulation were considered, from the point of view of discrete events and dynamical systems, as a tool for system modeling and analysis.

The advantages of modeling and analyzing systems using Petri nets are those generated by a model that can be used to analyze behavioral properties for the construction of systematic performance evaluation systems and control systems.

The graphics provided by Petri nets allow the visualization of system activities, improving system specifications to avoid complex mathematical notations, interpretation queues, errors, and possible accidents that may occur in the system.

This concept allows, from the design phase, the creation of an intelligent system using equipment that can track, record, display, monitor, and adjust parameters autonomously. For the cloud, we turned to the simple definition of the cloud as an application available only to customers or users with active mobile Internet that provides a solution for data storage. Cloud storage is about archiving, organizing, and distributing data on demand between virtualized storage volumes that have been consolidated in hardware.

Cloud activity is considered, according to the specialized literature, to be a processing paradigm and has recently received a lot of interest from researchers, gaining attention in the educational sector as well. This paradigm assumes that the modern educational system is currently divided into two major branches—global processing networks and analysis and interpretation chains—allowing the shared use of globally distributed processing systems and resources. The concept of cloud education is considered one of the main directions in the development of the educational system. Cloud education is an evolving paradigm in which dynamically scalable and virtualized processing resources, skills, and capabilities involved in the entire lifecycle are provided to users in the form of complex services. The basic building blocks of cloud education are modern technologies and cloud computing. Cloud processing is a solution through which users can request services, from information processing design, testing, and management to all other stages of the information lifecycle [2].

This paper tackles some empirical elements and is organized on several architectural levels that highlight aspects related to the concept of the cloud in the educational system. It also points out that the IoT system, with its advantages, is a system that is already a necessity for the evolution of modern technologies with implications in all stages of monitoring.

The introduction of cloud technologies in the intelligent information-processing systems of the future has become an important goal for education. Modern technologies for information-processing systems could have a major impact in terms of increasing the efficiency and availability of information-processing systems, implicitly leading to increased equipment efficiency and increased overall value. The aim of introducing intelligent information processing is to reduce costs, processing and delivery times, etc.

The introduction of technologies in intelligent information processing plays a key role, as it is desired to make these activities flexible and agile. This approach is a big challenge since it is the starting point for planning the information processing for the future and the introduction of new technologies into these activities [3].

The proposed architecture of the chosen model is adaptable to information processing and other processes and is intended to enable designers to create intelligent equipment, so that it can track, record, display, monitor, and adjust parameters autonomously.

**Figure 1:**
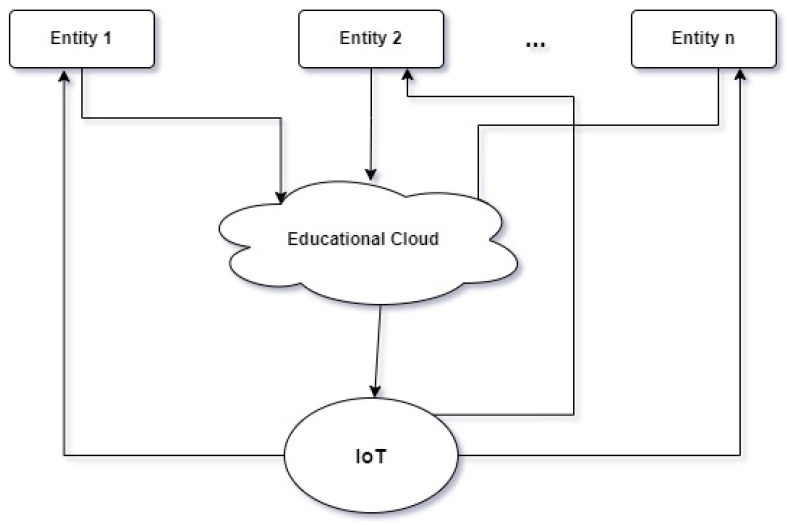
Architecture of the chosen model.

As can be seen in Figure 1, the architecture of our model proposes an educational system modeling method consisting of n entities, a cloud, and a private approach to IoT technologies.

Figure 1 highlights the elements of the proposed architecture. IoT refers to the multitude of IoT devices used by both students and experts in the teaching–learning–assessment processes. These IoT devices send relevant information to an educational Cloud. The structuring of information on the Cloud is carried out according to entities that are scientific fields studied in higher education (e.g., economics, engineering, computational linguistics, computer science, medicine, law, etc.). As presented in Section 4 of this paper, which deals with three different domains, the types of data we work with are entity specific. The cloud data can be accessed in real time using IoT devices by authorized people. 

To build a reference architecture, a literature review was carried out to decide a plan or framework that could provide current or future descriptions of a particular domain composed of components, and the interconnections, actions, or activities that those components perform. These activities, in turn, have strict rules and specific constraints.

According to field literature, a reference architecture identifies the functions required to achieve a set of objectives in each domain and the types of systems that perform or support the entities in performing the activities that implement those functions and outlines the nature and content of the required interfaces between those systems. The purpose of the reference architecture is to identify the activities and information flows required to perform the compulsory functions and to standardize those activities to ensure their effective performance [4].

The role of the reference architecture is to reduce system complexity as it anticipates and addresses important issues, allowing activities to accelerate model development and deployment.

The proposed architecture is applicable at the component, equipment, or information-processing method level. It can be implemented in flexible intelligent systems and equipment capable of interacting and communicating across hierarchical levels through a network.

This paper proposes a new architectural model of intelligent information processing, allowing specialists to perform simpler and optimized planning using key learning technologies.

The existing architecture model provides a good overview of the environmental architecture but leads to some limitations for users. To overcome these limitations, we proposed a simple model of intelligent information processing architecture based on the concept of distributed systems with accurate information and data flows between them. The proposed architectural model enables more reliable and simpler intelligent modeling. It provides the reference architecture model which is based on a three-dimensional map showing the problem approach at the educational level, in a structured manner, and last but not least, it ensures that participants obtain the desired information [5].

According to the literature [5], the integration of decentralized resources and the establishment of a collaborative infrastructure between entities of any kind is fundamental. This idea requires building a networked collaboration environment to integrate information processing resources and applications. Information processing resources and knowledge are brought into the cloud, and thus become accessible on consumer demand. Cloud services are readily available, location independent, and used to store and compute the data generated. Cloud services are most suitable for IoT systems due to various factors such as easy availability, managing resource constraints, and different policies. Cloud servers can provide services in the form of software, storage, computing power, and platforms.

## 2. Related Work

The extensive literature regarding the use of IoT with or without the use of a cloud in different systems shows the advantages of cloud manufacturing [5] and the use of an IoT educational system without the use of a cloud [6].

The economic literature offers a vast area of documentation on the importance of using electronic media to identify, collect, analyze, and process economic data and their use in economic practice, both in improving financial education and in increasing the performance of companies.

The business world is constantly changing due to the opportunities offered by information technologies in making business decisions. The implementation of technologies is essential to the development and efficiency of business.

Cloud computing as a business model is considered efficient [7] because it converts capital expenditures into operating expenses, thus facilitating the availability of financial funds for running current business operations.

The advance of digitization, the increasing use of technologies, the transfer of data, the increasing application of artificial intelligence in as many fields of activity as possible, and the Internet of Things are the determinants that transform society towards a sustainable development [8].

The study of languages using computer applications, automatic translation with the help of applications, and the use of applications in learning a new language or language modeling for IoT applications [9] has received a lot of attention in recent years, especially in the current context of globalization. 

The geodetic domain is open to the use of the newest devices for cadastral measurement, offering connection options for real-time transmission of information, which allows quick access for processing and analysis.

A very interesting paper about a prototype of IOT GNSS sensor for cloud GNSS signal processing is presented in [10].

## 3. System Theoretical Analysis 

IoT technology allows easy connection to IoT devices and real-time data collection and analysis to optimize the process quickly and make it as efficient as possible [11].

IoT is recognized for its benefits and advantages:

- Collecting reliable and secure data from devices.
- Integrating devices into the institution/company easily.
- Real-time performance of big data and predictive analysis of IoT flows and events.
- Extending applications and processes with IoT data.
- controlling devices with mobile applications.

Companies have trouble ensuring that the most efficient equipment operates over a long period of time.

Equipment maintenance costs have recently become an issue because of failures caused by older equipment, the association between old and new equipment, or due to system errors caused by some incompatibilities. Remote monitoring and maintenance that are offered by IoT represent solutions to these challenges. The IoT data integration allows reduced costs and also allows time to repair and monitor the flow, which are solutions to digital systems challenges [12].

The role of IoT as a major change is driven by harnessing data through data acquisition, transmission and storage, cloud-based tiered centralization, and analysis [12].

To start with, we focus on categories of functions provided by IoT systems.

Cloud systems are currently available from small to global systems. They are the focus of research due to their wide applicability. The use of cloud systems, be it for application deployment or part of the infrastructure, requires the understanding of the differences and benefits of cloud services. Three cloud service models were analyzed [13]: Software as a Service (SaaS), Platform as a Service (PaaS), and Infrastructure as a Service (IaaS). Each of these models has its own benefits and the awareness of their differences ensures an optimal choice.

SaaS as a service is the most commonly used option for cloud users. SaaS uses the internet to deliver applications managed by a provider. SaaS is delivered through a web browser and requires no downloads or installations on computers [13].

Software as a Service (SaaS) delivers predefined applications as a service over the Internet or distributed environment, which is a great advantage for the application analyzed in this paper [14].

With SaaS, distributors can manage all technical issues, such as data, middleware, servers, and storage, and the system chosen for analysis can simplify and maintain support.

The big advantage of SaaS is the time and cost reduction of software installation, management, and updating, which leads to staff reduction and distribution to other technical activities within the system.

Optimal conditions for using SaaS include:

- Quick e-commerce launch and zero time for servers and software;
- Short-term projects requiring collaboration;
- Use of less demanding epics;
- Applications requiring web and mobile access.

A major challenge for SaaS providers looking to exploit the benefits of cloud computing is to manage their engagements throughout the lifecycle of a service. The complexity of this problem led to the emergence of new offerings called PaaS that aim to abstract this complexity through specific tools and services [14].

PaaS as a service provides cloud components of certain programs while they are used for applications. PaaS provides a framework for developers to build and use custom applications.

Servers, storage, and networking are generally managed by the system or vendor, but developers manage the application [13].

PaaS wants to use a simple idea, even if the execution is complex, since many apps have a single development platform and common services, including authentication, authorization, and billing. PaaS developers build web applications without having to install tools on their computer and deploy these applications without having to know or care about the system complexity or managing the underlying hardware and software layers. A PaaS program is built on an IaaS platform and uses a powerful development and deployment tool [14].

The PaaS architecture aims to provide tools and techniques for modeling, simulation, analysis, planning, provisioning, and real-time monitoring of service-oriented applications, and virtual storage in the deployed cloud [14].

Platform as a Service (PaaS) can provide a platform for development and a special environment which can offer different services and storage, hosted in the cloud [14].

The PaaS hosting service must provide adequate resources to meet market requirements, along with adequate availability opportunities.

The PaaS provider supplies a suitable environment for general developers to build web applications without deep domain server expertise and website development or management.

The PaaS user must have a browser-based development environment, the ability to deploy a hosted environment, management tools, monitoring capabilities, and the ability to make payments at the time stipulated in the contract [14].

Features that define PaaS as a cloud service include:

- Resources can be reduced through virtualization technology;
- Services for application development, testing and deployment are provided;
- Same application can be accessed by multiple users;
- Web services and databases are integrated.

PaaS is useful if there are multiple developers working on the same project or if there are elements that need to be introduced since it can provide high speed and flexibility to the whole process. PaaS is useful for creating custom applications, and it can reduce the issues that arise in applications development and deployment costs.

## 4. Information Processing by Entities

This chapter focuses on the presentation of entities and how information processing takes place within them. Furthermore, the use and integration of smart devices and IoT devices in the teaching–learning process in higher education are highlighted.

The advance of technology in recent years, both in pre-university education and in university education, brought in amazing teaching techniques, integrating different smart devices as well as a multitude of computer applications. With the use of electronic devices as well as computer applications, teaching methods are much more varied, interactive, and much more attractive for pupils and students.

Interactive presentations, videos, educational games created for certain subjects and years of study, simulations and online experiences, team projects using different cloud computing applications, online tests, but also other methods and strategies of teaching can greatly raise the level of attractiveness and accessibility of a discipline. The teaching–learning–assessment relationship becomes dynamic and attractive. The evolution and improvement of teaching methods and techniques using different IoT devices as well as different IT applications can lead to changes in the act of learning.

A dynamic and pro-active teaching process as well as instant student access to correct and complete information available on dedicated educational cloud increase motivation and the training quality. 

Higher education institutions use various IoT devices in the learning–teaching–assessment process successfully for the benefit of students and teachers, among which smart boards, tablets, audio and video recording devices, virtual and augmented reality devices, as well as different sensors and monitoring devices are used to collect certain data of interest. Nowadays, most young people own smartphones or tablets that they use to access different educational materials or to collaborate with peers to prepare assignments or team projects.

According to the literature [15], decision making within the company is based on reason, analysis of available data, digital solutions, and calculations, which is applicable to any company or institution as long as continuous access to data is provided. 

The choice of the three entities addressed and presented in this paper is not accidental, aiming to cover different domains working with different types of data, and automatically with different data processing. The three domains, economics, computational linguistics, and engineering are very different in terms of specific approaches and challenges. Teachers in these fields choose and adapt their teaching methods and techniques according to the subject and to the specific objectives. Consequently, different devices may be used, different types of information processing are carried out, and different types of processed data are uploaded to the cloud.

In the following sub-sections, different examples of information processing on each entity create an overview of the types of data processed within the proposed system entities.

### 4.1. Economics

The Internet of Things (IoT) has introduced ground-breaking changes to the methods used to gather and examine data, as well as brand-new opportunities for individuals and companies alike. In the field of economics, the Internet of Things (IoT) may be used to gather and examine economic data such as Gross Domestic Product (GDP), inflation rate, interest rate, and unemployment rate. Students may be educated using the data to learn about the relationships between various economic indicators and the ways in which these indicators impact the economy. This section of the article covers the process of plugging a software program that can load data from a public source like Eurostat into the cloud and use it to teach students about economic statistics.

#### 4.1.1. 1st Example

The gathering of data is the initial task involved in using IoT for the purpose of economic data analysis. Eurostat is widely regarded as one of Europe’s most credible resources for economic statistics. The Gross Domestic Product (GDP), the Inflation Rate, the Interest Rate, and the Unemployment Rate are only a few of the economic statistics that may be obtained from Eurostat and utilized for research purposes. Below, we have described the process of automatically accessing data from Eurostat, downloading it from the website, and loading it into the cloud.

1. Create an account with Eurostat: In order to gain access to the Eurostat Application Programming Interface (API), one will need to create an account on the Eurostat website by filling out the registration form with the required information.
2. Obtain an API key: After successfully creating an account, one needs to obtain an API key in order to gain access to the Eurostat API. The API key is a one-of-a-kind identifier found on the Eurostat website. Following the procedures of the provider, one will be able to generate an API key from the specific location.
3. Select the information that you wish to have access to: The Application Programming Interface (API) for Eurostat gives users access to a large array of economic statistics, such as gross domestic product, inflation, interest rates, and unemployment rates. Once the data have been identified and localized, one needs to decide on the data format needed for the platform to be able to use it in dashboards, analysis, and reporting. Eurostat provides its users with access to a variety of data formats, such as CSV, JSON, and SDMX.
4. Once the APY key has been obtained, one can automatically connect to Eurostat data. One of the tools that can be used is Apache NiFi, a well-known software application that is used for connecting to APIs in general. The NiFi platform is open-source software that enables users to automate the transfer of data from one system to another.

The following procedures need to be carried out for you to connect NiFi to the Eurostat API:

1. Install the ETL tool Apache NiFi. To be able to access data from Eurostat API, one needs to install the tool Apache NiFi to the PC that will perform the automatic process. The ETL tool will be able to generate a data flow through the “InvokeHTTP” processor to be able to access the Eurostat API.
2. Configuration of InvokedHTTP. In order to configure the InvokeHTTP processor, one needs to input the URL of the Eurostat API, as well as the API key and any other parameters that are necessary to have access to the desired data. Additionally, one needs to specify the format in which you want the data to be delivered.
3. Execute the data flow. Once the configuration of InvokeHTTP processor is completed, the system is ready to execute the data flow. NiFi will establish a connection to the Eurostat API, obtain the data requested, and save it in a manner that may be utilized for additional analysis.

When the data have been transferred to the cloud, users have access to a variety of tools that allow them to construct dashboards, generate reports, and conduct OLAP (Online Analytical Processing) analysis. Tableau is a widely used application for the creation of dashboards. Tableau is a data visualization application that can be used to generate interactive dashboards that present economic data in an understandable manner. These dashboards can be used to analyze and make decisions based on the data. UsingTableau allows users to generate dashboards that display the relationships between GDP, inflation rate, interest rate, and unemployment rate.

Power BI is another well-known tool for the creation of reports. Power BI is a business analytics tool that allows users to generate reports that meaningfully present economic data. You can generate reports that demonstrate the links between GDP, inflation rate, interest rate, and unemployment rate by using Power BI.

#### 4.1.2. 2nd Example

The Internet of Things system could be used to collect data on soil humidity, air humidity, temperature, and precipitation, which could be a game-changer for agriculture since it enables farmers to have a better understanding of the needs of their crops and to optimize their irrigation system, which ultimately results in higher yields and more efficient use of resources. In this example, we will cover the process of plugging in software that can load data into the cloud through Internet of Things sensors. These data may then be used to adjust soil irrigation in order to maximize output.

Step 1: Determine the objectives and the prerequisites.

It is vital to outline the goals and criteria of any Internet of Things project before beginning the work. In this particular scenario, the main goal is to gather information on soil humidity, air humidity, temperature, and precipitation to maximize irrigation efficiency and crop output. Because of this, the following terms need to be defined:

One needs sensors to gather data on soil humidity, air humidity, temperature, and precipitation. In addition to this, it is important to estimate the needed quantity of sensors as well as the characteristics of those sensors, such as their accuracy, range, and power needs.

The communication protocol: It is necessary to select a communication protocol that will enable the sensors to send data to the cloud. This might be a low-power wide-area network (LPWAN) like LoRaWAN [16,17] or SigFox [18], or it could be something like Wi-Fi or Bluetooth. In addition to this, one needs to make sure that the cloud platform can interface with the software that will be utilized to evaluate and calibrate the irrigation system.

Step 2: Pick the appropriate software and hardware.

The Internet of Things system’s hardware and software components need to be chosen depending on the desired outcomes and prerequisites. The following are required for this project:

- Sensors: In order to obtain information on temperature, precipitation, soil humidity, and air humidity, the project requires sensors. Rain gauges, soil moisture sensors, temperature and humidity sensors, and other types of sensors are just examples of the many types of sensors needed. It is imperative that the selected sensors are compatible for use with the communication protocol selected and are able to deliver data that are accurate and in real time.
- Microcontroller: To gather information from the sensors and send it to the cloud, a microcontroller needs to be used. The microcontroller must have sufficient processing power and memory to be able to deal with the data, in addition to being compatible with the communication protocol.
- Communication module: A communication module is required that enables the microcontroller to send data to the cloud to complete this project. Depending on the communication protocol that has been decided upon, this component may be a Wi-Fi module, a Bluetooth module, or an LPWAN module.
- Platform hosted on the cloud: The cloud platform should be able to collect, store, and process the data that are gathered by the sensors. A wide variety of cloud systems are currently accessible, including Amazon Web Services (AWS), Microsoft Azure, and Google Cloud Platform, amongst others. One needs to decide on a platform that has the capability to integrate with the program that will be utilized to evaluate and calibrate the irrigation system.
- Software for the Internet of Things: The software should be able to load data from IoT sensors into the cloud and utilize these data to adjust soil irrigation to maximize production. ThingWorx, IBM Watson IoT, and Microsoft Azure IoT are just a few of the Internet of Things software platforms that are now available. It is necessary to select a software platform that can integrate with the cloud platform chosen and that has the requisite features and capabilities.

Step 3: Hardware setup

After deciding on the various components of the hardware, the next step is to set it up. The following are requested during hardware configuration:

Establish a connection between the sensors and the microcontroller.

It is necessary to establish a connection between the sensors and the microcontroller and verify that they are operating appropriately. Connect the communication module to the microcontroller: In order to complete this step, the communication module needs to be connected to the microcontroller and it must be verified that the two devices are able to communicate effectively with one another. A source of power must be supplied to the microcontroller as well as the communication module before the system is turned on.

Certain steps must be followed depending on hardware configuration. 

Configure the microcontroller: To read data from the sensors and send it to the communication module, the microcontroller needs to be set properly. This includes programming the microcontroller, establishing any necessary drivers or libraries, and setting up the input and output pins.

Step 4: Configuring the Cloud

Once the hardware has been installed and validated, the cloud platform should be installed and configured. The following components make up the cloud infrastructure:

- Establish an account: To use the cloud platform, first, an account needs to be established with it and then configured with the appropriate rights and security settings.
- Set up the Internet of Things service: In order to receive and store data from the sensors, the Internet of Things service that is supplied by the cloud platform needs to be set up. During this step, any necessary security settings need to be configured, the communication protocol needs to be set up, required data structures should be created, and so on.
- Perform some tests on the cloud platform: Once the cloud computing platform is set up, it needs to be tested to ensure that it is capable of correctly receiving and storing data, as well as providing the necessary features and functionality.

Step 5. Setup of the IoT software

In the end, the IoT software needs to be configured so that it can upload data to the cloud via IoT sensors and then utilize those data to calibrate the soil irrigation system to enhance production. The installation of the IoT software includes the following steps:

- Install the program: To access the cloud platform, the IoT software needs to be installed on a computer or server that has network connectivity.
- Configure the software: To connect to the cloud platform and retrieve data from the sensors, the software must be configured so that it can be used. In addition to this, the software must be configured so that it may analyze the data and provide recommendations for the irrigation calibration.
- Test the program: After the software is installed, it should be checked that it successfully retrieves data from the sensors, accurately analyzes the data, and provides the required suggestions for irrigation calibration.

Step 6. Integration

The final stage in the process is to establish a seamless system by integrating the IoT software, cloud platform, and hardware components. The following are components of the integration:

One needs to configure the cloud platform so that it may transmit data to the IoT software and obtain recommendations for irrigation calibration, which will allow users to complete the connection between the cloud platform and the IoT software.

In conclusion, in order to gather data on soil humidity, air humidity, temperature, and precipitation, it is necessary to create a special system, which requires careful planning and implementation. During this phase of the process, determine the goals and requirements, select the hardware and software components, set up the hardware and cloud platform, configure the IoT software, and finally integrate the system. An Internet of Things system has the potential to be a useful tool for farmers, providing them with the ability to enhance resource utilization, boost production, and optimize irrigation, when it is implemented properly.

The literature indicates that companies adopt new digitization tools aware of the opportunities that their use offers in their approach to market growth, expanding the number of customers, offering new services on the market, reducing costs, all this influencing the management system of the organization [19].

### 4.2. Computational Linguistics

Computational linguistics is an interdisciplinary field that deals with the study of natural language using various computer science techniques (e.g., NLP, AI, statistics, etc.).

Computational linguistics is a discipline of interest for both computer scientists and philologists. If linguists use already created applications for the study of natural language, computer scientists focus on improving the applications and creating new applications according to the specifications desired or presented by the linguists so that they are useful to the latter in the language study.

Numerous computer applications as well as different types of mobile devices are used in the teaching–learning–assessment process. The following subsections present specific examples of mobile devices and IoT devices as well as different computer applications use, data processing using these applications, data processing results, and data that are accessible for extraction or query using the Education Cloud.

#### 4.2.1. The Process of Teaching and Learning a New Language

Within the framework of university programs in Foreign Language and Literature or Translation and Interpreting, new languages are often taught to beginners, where the emphasis is on vocabulary and grammar, pronunciation, and spelling.

There are steps that must be followed by students to learn new languages during their university education. These steps are known by the teachers and most of the time they are presented in the first lesson:

- The clear establishment of the requirements, rules, and objectives of the discipline so that the students remain motivated throughout the semester/year.
- The choice and communication of all teaching methods used during the course: physical teaching can be combined with individual study using different on-paper materials or other online resources, maybe even different IT applications.
- Vocabulary building starts with learning the most frequently used words or sentences, and specific teaching methods can be established: the use of flashcards, the use of visual presentations considering that it is proven that information is much easier to remember when presented in visual form, the use of computer applications open to the general public, etc.
- Practicing pronunciation with the help of specific exercises during class hours, watching different video resources prepared by the teacher, and repeating dialogues or using different computer applications that offer a pronunciation check, such as the Mondly application.
- Learning grammatical and syntactical rules is mandatory. These rules must be deepened and practiced both within the course and seminar hours and individually, solving certain exercises.
- Practicing the materials reading and understanding the audio and video materials. It is recommended to read books and specialized articles and listen to or watch certain recommended materials to improve understanding and increase vocabulary.
- Practicing writing and speaking through various mandatory exercises such as writing essays on a certain topic or carrying out dialogues or discussions, in class, under the supervision of the teaching staff on a certain topic.

#### 4.2.2. Examples of Exercises That Can Be Applied in the Process of Teaching and Learning a New Language

First Example: Vocabulary enrichment.

Vocabulary enrichment is very important in second language acquisition [20] and starts from the first hour of new language learning. Usually, the notions of interest are grouped by categories: greetings, numbers, colors, fruits, vegetables, household items, clothing items, places, times of day, etc. The list can continue with the most common verbs, pronouns, simple sentences, etc. For each of these notions, images contain the object or information, the name in the native language, and the name in the language that is desired to be learned. Images can also be made by category in .jpg or .png format, maybe even small .gif animations that capture all the objects/information/actions in that category.

Students can receive team projects to create images for new categories.

The respective images, both those made available by the teaching staff and those made by the students, can be uploaded to the educational cloud, searched for later, and accessed at any time by the students or evaluated by the teaching staff.

Second Example: pronunciation practice.

Pronunciation is vital in the process of learning a new language, which accounts for its special place in the student evaluation process.

Practicing pronunciation improves speaking skills and leads to increased speech clarity [21]. In seminars, pronunciation exercises and reading aloud exercises are used to observe and correct pronunciation. Unfortunately, only these exercises performed in an organized setting are not enough. This is where individual study and home exercises come into play. It is known that in the case of this type of exercise, quick feedback is needed, which, in the case of individual study, cannot be given by a teacher. In this situation, the teacher can recommend the use of computer applications, such as Mondly.

Mondly is a very well-known computer application dedicated to the study of foreign languages. It offers a series of functions, including pronunciation exercises. To use this type of exercise, the microphone on the device on which the application is installed should be functional and activated. The desired exercise is selected, and the words or sentences displayed on the screen are read aloud. With the help of voice recognition, immediate feedback on the exercise performed can be received.

For this type of exercise, audio (MP3 or WAV, etc.) and video (MP4, MOV, AVI, WMV, etc.) materials can be uploaded to the cloud, which the student must practice. Various documents, .docx, .txt or .pdf type articles that should be read aloud as an exercise can also be uploaded to the cloud.

Students may receive individual or team projects asking them to read, translate, and read a piece of text. The reading must be recorded and uploaded to the cloud. It can be accessed and/or downloaded to be evaluated or to be discussed and analyzed in class.

Third Example: Translating a text.

Translating words from one language to another is easy and requires knowledge of the words. Everything changes when a text needs to be translated, since students must have knowledge of vocabulary, syntax, semantics, grammar, pragmatics, topics, punctuation, spelling, and more. Students need to understand the meaning of the text in their mother tongue because it should not change through translation.

As translation exercises in the classroom, it is recommended to choose relevant texts that can be well understood by the students and built on their already acquired knowledge [22].

It is also recommended to progressively increase the difficulty of the texts, from simple to complex, slowly opening the way to figurative language, as students learn figures of speech and manage to recognize them.

During the exercises, using the smart board, different documents containing the text to be translated and the text translated together in class can be created. Certain elements (such as different parts of speech or sentences, depending on the teacher’s objectives) are to be highlighted in different colors. Also, the apps allow comments to be entered. Annotation exercises can be carried out in seminars where different stylistic figures can be approached and discussed, respecting certain rules [23]. These documents are uploaded to the cloud with representative names so that they can be found and accessed by students easily.

Learning a new language is a complex process, and higher education teachers who teach such a discipline want their students to master the newly learned language as well as possible. The teachers aspire that their students understand and study the new language in depth and therefore, often, in the exercises carried out together, they also use different special applications such as Concordances, Machine Translation, spell and grammar checking, chatbots, knowledge extraction tools, etc. All the results of these exercises can be inserted into certain documents that can later be uploaded to the cloud and accessible for later access.

### 4.3. Engineering

The interest in Satellite Geodesy has increased recently, as it is included both in the educational plans of the Land Measurements and Cadastre specialization, but also in those of other specializations such as Environmental Engineering, Urban Engineering and Regional Development, Cadastral Information Systems and Real Estate Management, etc.

The discipline of Satellite Geodesy (also known as Space Geodesy or Satellite Geodetics) involves the use of satellite-based technologies to measure and understand the Earth’s shape, orientation, gravity field, and other physical parameters.

Internet of Things (IoT) and Global Positioning System (GPS) technologies are both rapidly evolving and changing the way we interact with the world around us.

This paper presents the steps taken to acquire the data using GPS technology, and the integration of the data into a Cloud to be accessed by students, through IoT, for the purpose of processing and interpreting data.

These technologies have great importance in educating students, as they are increasingly being used in various industries and offer numerous opportunities for innovation and development.

#### 4.3.1. GPS and IoT

GPS (Global Positioning System) and IoT (Internet of Things) are two different technologies that have the potential to work together and create new opportunities for education and businesses.

GPS (Global Positioning System) is not an IoT (Internet of Things) device in itself, but it is often used as a component of IoT devices or systems.

IoT devices typically consist of a network of interconnected devices that collect, transmit, and process data to achieve specific objectives [24]. These devices are usually equipped with sensors, processors, and communication modules that allow them to interact with other devices and with the cloud.

GPS, on the other hand, is a satellite-based navigation system that provides accurate positioning and timing information to users with GPS receivers [25]. GPS devices are not usually equipped with sensors or communication modules, but they can be used in combination with other sensors and communication devices to provide location-based data to IoT applications.

For example, GPS can be used in combination with other sensors such as temperature, humidity, and pressure sensors to monitor environmental conditions in real time. The location data obtained from GPS can be transmitted to the cloud through a communication module such as cellular or satellite communication, allowing IoT applications to process and analyze the data to achieve specific objectives.

The principle of GPS real-time measurements involves using a GPS receiver to measure the signals transmitted by GPS satellites in real time and calculating the position of the receiver based on these measurements [26].

#### 4.3.2. The Components and Peripherals of a Permanent GNSS Station

The basic equipment and peripherals of a permanent GNSS (Global Navigation Satellite System) station include:

- GNSS Receiver: The GNSS receiver is the main component of the permanent GNSS station, which receives signals from GNSS satellites and records the observations for post-processing.
- Antenna: The antenna is a critical component of the GNSS station, which collects the signals from GNSS satellites and passes them to the GNSS receiver.
- Data logger: The data logger stores the observations recorded by the GNSS receiver for later analysis.
- Power supply: The GNSS station requires a reliable power supply, which can be AC or DC depending on the location and power availability.
- Communication peripherals: The GNSS station requires communication peripherals to transmit the data from the station to the control center or data center. These peripherals may include a modem, router, radio modem, or satellite modem.
- Protection enclosure: The GNSS station is typically installed in an enclosure, which protects it from weather or damage in case of accidents.

Communication with the receivers in the GNSS station can be achieved through various communication peripherals, depending on the type of station and the available communication infrastructure [27].

The most frequently used is Internet communication. The GNSS station is connected to a modem, which transmits the data to a data center through the internet network.

To connect a GPS receiver to permanent stations using the internet, you need a GPS receiver that is compatible with internet connection and software that can connect to the permanent stations.

#### 4.3.3. The Connection of the GPS Receiver to Permanent Stations Using the Internet

To connect the GPS receiver to permanent stations using the internet involves the next steps:

- Checking the compatibility of the GPS receiver to ensure that the GPS receiver is compatible with an internet connection and has communication ports available to be connected to the internet, such as USB port or serial port.
- Configuring the GPS receiver to connect to the internet may vary depending on the type of GPS receiver but usually involves entering connection information such as an IP address and port.
- Downloading and installing the software for connecting to permanent stations through the internet.
- Connecting the GPS receiver to a computer or network device using the USB or serial port.
- Configuring the software to connect to permanent stations and transmit the data through the internet.
- Verifying the connection between the GPS receiver, the connecting software, and the permanent stations is working correctly, and that data are being successfully transmitted.

#### 4.3.4. GPS Real-Time Measurements

The principle of GPS real-time measurements involves using a GPS receiver to measure the signals transmitted by GPS satellites in real time and calculating the position of the receiver based on these measurements.

The steps of GPS real-time measurements are as follows:

- Satellite signals transmission: GPS satellites transmit signals that travel at the speed of light to the GPS receiver on the ground.
- GPS receiver measurement: The GPS receiver receives the signals and measures the time it takes for the signals to travel from the satellite to the receiver. The receiver also measures the satellite’s location in space and the location of the receiver on the ground.
- Calculation of distance: Based on the time it takes for the signal to travel from the satellite to the receiver, the receiver calculates the distance between the satellite and the receiver.
- Trilateration: By measuring the distance to at least four GPS satellites, the GPS receiver can determine the position of the receiver on the ground through a process called trilateration.
- Real-time updates: The GPS receiver continuously updates its position as it receives signals from additional GPS satellites. This allows for real-time tracking and navigation.
- Error correction: To improve the accuracy of GPS measurements, various error correction techniques can be used, such as differential GPS (DGPS), which uses a reference station with a known location to correct errors in the GPS measurements.

#### 4.3.5. Real-Time Processing and Post-Processing

Real-time processing and post-processing are two different methods of GPS data processing.

Real-time processing involves processing GPS data as they are being collected in real time. The GPS receiver calculates the position of the receiver on the ground using the signals received from the GPS satellites and outputs the position information in real time.

Post-processing, on the other hand, involves processing GPS data after collection. GPS data are typically collected in a data file, and post-processing involves analyzing the data file to more precisely calculate the points/objects position.

Post-processing can involve various techniques to improve the accuracy of the GPS measurements, including differential GPS (DGPS) which uses a reference station with a known location to correct GPS measurements errors.

The data formats downloaded from GPS can vary depending on the type of collected data and the type of GPS device.

The common data formats downloaded from GPS are:

- NMEA 0183: This is a standard data format used for exchanging GPS data between different GPS devices. It includes information such as latitude, longitude, altitude, time, and speed.
- GPX: This is a file format used to exchange GPS data between GPS devices and software applications. It contains information such as waypoints, tracks, and routes.
- KML: This is a file format used to display GPS data in 2D or 3D formats in Google Earth or other GIS software. It includes information such as latitude, longitude, altitude, and time.
- RINEX: This is a standard data format used for exchanging GPS data between different GPS devices and software applications. It includes information such as raw GPS data, carrier phase measurements, and satellite ephemeris data.
- Shapefile: This is a file format used to store and exchange geospatial data in GIS software. It includes information such as points, lines, and polygons, as well as attribute data.
- CSV: This is a simple data format used to store and exchange tabular data. It includes information such as latitude, longitude, altitude, and time, as well as any additional attribute data.

#### 4.3.6. Integrating GPS Data into a Cloud

Integrating GPS data into a cloud-based platform can provide a powerful tool for educational purposes. Here are some steps to consider when integrating GPS data into a cloud-based platform for educational purposes:

- Identifying the educational objectives: The first step is to identify the educational objectives you want to achieve. Determine what skills or knowledge you want students to develop using GPS data.
- Choosing a cloud-based platform: Choose a cloud-based platform that meets your educational objectives and requirements. Popular platforms include Google Cloud, Microsoft Azure, and Amazon Web Services.
- Collecting GPS data: Collect GPS data using a GPS device or smartphone app. Ensure that the data are in a format that can be easily imported into the chosen cloud-based platform.
- Importing data into the cloud: Import GPS data into the cloud-based platform. Depending on the platform, this may involve uploading files, connecting to an API, or using a third-party tool.
- Visualizing the data: Use the cloud-based platform’s visualization tools to create maps, graphs, or other visual representations of the GPS data. This can help students understand the patterns and trends in the data.
- Analyzing the data: Use the cloud-based platform’s analysis tools to identify patterns and trends in the GPS data. This can help students develop critical thinking and problem-solving skills.
- Creating learning activities: Use the GPS data and the cloud-based platform to create learning activities that engage students and reinforce educational objectives.

Some potential educational uses of GPS data in the cloud could include studying migration patterns of animals, analyzing changes in land use over time, or exploring the impact of climate change on sea level rise.

By integrating GPS data into a cloud-based platform and using it for educational purposes, students can develop critical thinking skills, problem-solving skills, and a deeper understanding of the world around them.

This chapter highlighted and presented the fact that, depending on the entities considered, the data uploaded to the Cloud can be of different types because of the different particularities of each domain. Although the data that each entity work with are distinct and specific, the functionality of the process and of the system is the same.

## 5. System Overview

This paper presents a method for modeling an educational system consisting of three entities, a cloud, and a private approach to the IoT technologies. To model our system, we used Visual Object Net++, a Petri Nets modeler tool that includes a custom graphical user interface for functional block models. Using this application, our system was automatically translated into a hidden Petri net model. The effectiveness and reliability of an information processing system can be assessed once further numerical analysis or simulation measures of the discrete events of interest are performed. The architecture was explained briefly and presented together with a small application example. All described techniques were implemented as an extension of the Visual Object Net++ tool.

Petri nets are considered from the point of view of dynamic systems with discrete events as a tool for system modeling and analysis. The advantages of modeling and analyzing systems using Petri nets are that the model generated can be used to analyze behavioral properties for system performance evaluation for systematic control system construction. The graphs provided by Petri nets allow visualization of system activities, improving system specifications to avoid complex mathematical notations, interpretation queues, errors, or possible crashes that may occur in the system. Currently, Petri nets are widespread in many applications and are used in various fields, business process modeling, because they have a very accessible graphical representation and well-defined semantics that allow a formal analysis of the behavior and properties of the modeled systems [28]. The graphical description of Petri nets is easy to understand and very useful in describing natural systems as distributed in real time.

**Figure 2:**
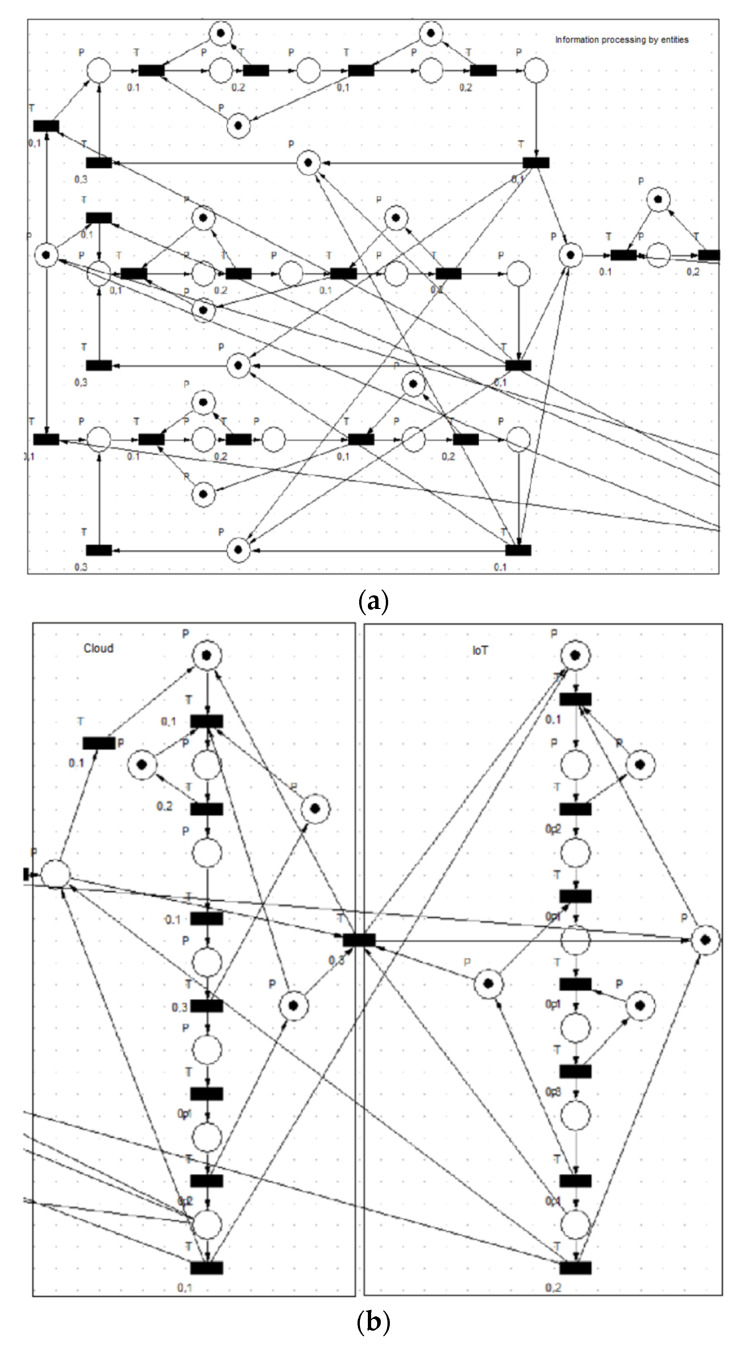
General model of the simulator (**a**,**b**) (generated using Visual Object Net++).

The information processing method is an empirical modeling of a complex system, to which one can easily adapt depending on the technology referred to in the practice. The paper is a description of a possible empirically analyzed model. Figure 2 shows times based on previous experiences, where each system was taken separately, studied, analyzed, and interpreted. 

The results obtained from the graphical representations are grouped by the three sectors under analysis: information processing by entities, analytics, and presentation, and representative charts were selected for each sector. 

Two sets of diagrams are shown in the figures, depending on the speed of work for modeling the system. We have not chosen diagrams from the information processing method because it is considered an ideal flow in which there are no significant fluctuations in the information transport system that is shown in the diagrams. There is a constancy for the beginning elements of the technological process, but there is a large variation for the information to be analyzed as seen in the previous section.

From the resulting diagrams below, it can be seen that there is an intense activity of information processing for the three entities analyzed. The flow of processed information is highlighted in these diagrams.

**Figure 3:**
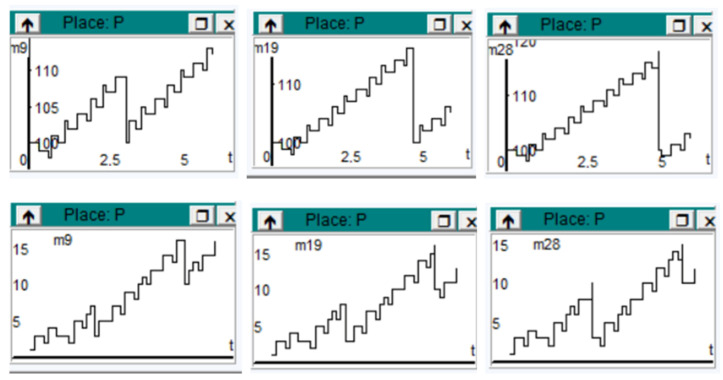
Diagrams for information processing, on two levels, depending on the speed of work. Level 1 coincides with line 1 (high speed) and Level 2 coincides with line 2 (lower speed).

Figure 3 shows the variations of information processing per entity. The diagrams are arranged on two levels. Level 1 provides three representations depending on the speed of work and level 2, being at a lower speed, shows the variation in the information flow during the processing time more precisely.

All the presentations of the diagrams are highlighted according to the two levels considered from the beginning of their analysis.

**Figure 4:**
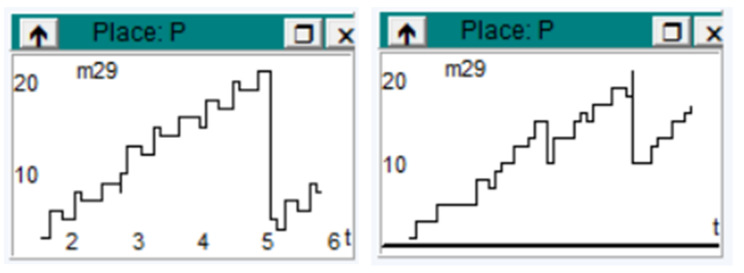
Diagram on completion of information processing.

Figure 4 shows the information load flow in the cloud. Again, a high level of activity can be observed.

**Figure 5:**
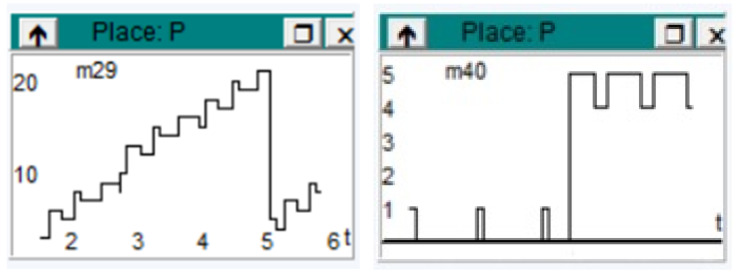
Diagram with information flow in the cloud results after system modeling using Visual Object Net++.

The figure represents the flow of information from the cloud and IoT. A correlation can be seen between the request for information with IoT devices and the response received from the cloud (Figure 5). The variation in the flow of information between entities is indicated. The flow is determined by information transport times, when the query flow is high and the information response from the cloud is high, and vice versa.

Regarding the study of systems in different domains such as manufacturing, industry, embedded systems, and process control, using Petri Nets has been of great interest in the research carried out in recent years, mainly due to the advantages they offer [29,30].

Also, the continuous development of IoT devices and the interaction between them brings into the research area various methodologies based on models and services for the integration of IoT systems or platforms in different areas [31,32].

The research is complex because it can combine, as in the case of the present research, the two topics of interest: Petri nets and IoT.

## 6. Conclusions

This paper presented an architecture and technique for modeling educational systems. We proposed the use of functional blocks for this application area, which is easier for users to understand than more formal description languages such as Petri nets.

The resulting models were automatically translated into a graphical Petri net model. After analyzing and simulating discrete events, the necessary actions can be taken for any point of interest. The performance and reliability of an information processing system can thus be evaluated.

Each element of our system had its well-established importance and role in the entire proposed model. The role of IoT devices was very clear in this whole process. IoT devices can be very useful when collecting data and information. Companies and institutions can obtain real-time data on their operations, as well as customer behavior and product demand in the specific market using sensors and other devices connected to the internet.

Practical evidence confirmed that IoT can change the way businesses operate. As a result, businesses may clearly and significantly increase their bottom line by implementing linked devices in business processes.

Within the entities, the information was processed and sent to the cloud. It can later be searched, accessed and/or downloaded.

An educational system allowing the use of IoT devices and storage of information on the cloud improves communication and collaboration between students and teachers. The use of such a system has other positive side-effects, such as the efficient management of teaching information resources for each subject area and free access to information, regardless of the time and place. 

For example, in the context of economic information, the cloud is very important because it allows companies to store and access data efficiently and in real time, thus enabling better business management and increasing the efficiency of their business.

So, the cloud is essential for information, providing companies or institutions with accessibility, efficiency, security, and the ability to analyze and access information in real time.

The model proposed in this work is perfectly applicable in higher education. IoT devices are increasingly accessible. Even if this paper referred to only three entities, it is possible to work with n such entities (Figure 1), where each entity can be a field or a department from a higher education institution.

The contributions presented in our paper should be of wide interest, especially for higher education. The system proposed and later modeled with the help of the Visual Object Net++ application can be successfully implemented in universities, regardless of their size. Since the system can be structured on n entities, it is flexible to be adapted to n fields, regardless of the specifics of the educational institution. The frequent use of IoT devices by students and teachers makes it possible to connect in real time to the educational cloud to upload information and access/search for or download data. It is difficult to predict how the impact of these technologies will shape the educational system in the future, which is why the dawn of cloud computing is now seen as a pinnacle of informational technology.

A team of researchers of the “1 Decembrie 1918” University of Alba Iulia is currently considering the perspective to create such a system and work on it. 

In the future, if such a system is to be implemented, we believe that a careful analysis of the types of data being worked with, analyzed, and then uploaded to the cloud is necessary. Each higher education institution should consider its own specificities; consequently, the data that can be collected with the help of sensors can vary considerably depending on the study field. In this case, new study opportunities open up for effective testing of the system. This creates new research opportunities that can focus directly on testing under specific conditions, depending on the application domain.

From our point of view, the limitations of such a system come from the external environment: access to the internet, the obligation to use IoT devices, and the obligation to receive access to the system. The security should be carefully considered as well. A very sensitive point to be considered is the security of the teaching data that go into the system.

Future research should further study the reliability of this system if the number of entities is very large, which involves secure management and use of a large volume of data of different types.

We agree that in the current context of business, political, and economic development, IoT is a priority that represents an essential element of their innovation strategy. It is essential that governments actively encourage the creation of a strong and durable IoT infrastructure and efficient structuring of data, ensuring fast and safe accessibility.

## Data Availability

Data sharing is not applicable.
